# The *Arabidopsis arc5* and *arc6* mutations differentially affect plastid morphology in pavement and guard cells in the leaf epidermis

**DOI:** 10.1371/journal.pone.0192380

**Published:** 2018-02-21

**Authors:** Makoto T. Fujiwara, Mana Yasuzawa, Kei H. Kojo, Yasuo Niwa, Tomoko Abe, Shigeo Yoshida, Takeshi Nakano, Ryuuichi D. Itoh

**Affiliations:** 1 Department of Materials and Life Sciences, Faculty of Science and Technology, Sophia University, Chiyoda, Tokyo, Japan; 2 Nishina Center and Plant Functions Laboratory, RIKEN, Wako, Saitama, Japan; 3 Laboratory of Plant Molecular Improvement, Graduate School of Nutritional and Environmental Sciences, University of Shizuoka, Suruga, Shizuoka, Japan; 4 Gene Discovery Research Group, Center for Sustainable Resource Science, RIKEN, Wako, Saitama, Japan; 5 CREST, JST (Japan Science and Technology Agency), Kawaguchi, Saitama, Japan; 6 Department of Chemistry, Biology and Marine Science, Faculty of Science, University of the Ryukyus, Nishihara, Okinawa, Japan; NARO Institute of Agrobiologial Sciences, JAPAN

## Abstract

Chloroplasts, or photosynthetic plastids, multiply by binary fission, forming a homogeneous population in plant cells. In *Arabidopsis thaliana*, the division apparatus (or division ring) of mesophyll chloroplasts includes an inner envelope transmembrane protein ARC6, a cytoplasmic dynamin-related protein ARC5 (DRP5B), and members of the FtsZ1 and FtsZ2 families of proteins, which co-assemble in the stromal mid-plastid division ring (FtsZ ring). FtsZ ring placement is controlled by several proteins, including a stromal factor MinE (AtMinE1). During leaf mesophyll development, *ARC6* and *AtMinE1* are necessary for FtsZ ring formation and thus plastid division initiation, while *ARC5* is essential for a later stage of plastid division. Here, we examined plastid morphology in leaf epidermal pavement cells (PCs) and stomatal guard cells (GCs) in the *arc5* and *arc6* mutants using stroma-targeted fluorescent proteins. The *arc5* PC plastids were generally a bit larger than those of the wild type, but most had normal shapes and were division-competent, unlike mutant mesophyll chloroplasts. The *arc6* PC plastids were heterogeneous in size and shape, including the formation of giant and mini-plastids, plastids with highly developed stromules, and grape-like plastid clusters, which varied on a cell-by-cell basis. Moreover, unique plastid phenotypes for stomatal GCs were observed in both mutants. The *arc5* GCs rarely lacked chlorophyll-bearing plastids (chloroplasts), while they accumulated minute chlorophyll-less plastids, whereas most GCs developed wild type-like chloroplasts. The *arc6* GCs produced large chloroplasts and/or chlorophyll-less plastids, as previously observed, but unexpectedly, their chloroplasts/plastids exhibited marked morphological variations. We quantitatively analyzed plastid morphology and partitioning in paired GCs from wild-type, *arc5*, *arc6*, and *atminE1* plants. Collectively, our results support the notion that ARC5 is dispensable in the process of equal division of epidermal plastids, and indicate that dysfunctions in ARC5 and ARC6 differentially affect plastid replication among mesophyll cells, PCs, and GCs within a single leaf.

## Introduction

Chloroplasts are double membrane-bound organelles that form a homogeneous population with respect to shape (round or ellipsoidal) and size (usually 3–10 μm in diameter) in photosynthetic cells. Such homogeneity in chloroplast shape and size is attained *via* several cycles of symmetric binary fission of the organelle, which eventually generate up to several hundred chloroplasts per cell. The most popular model for studying chloroplast division in higher plants is leaf mesophyll cells [1–3]. The discovery of *Arabidopsis thaliana* chloroplast number mutants (*accumulation and replication of chloroplasts*; *arc*), which was the first successful example of mutant screening to identify subcellular structure mutants in plants, was achieved through the observation of leaf mesophyll cells [4]. Among the *arc* mutants, the recessive *arc5* and *arc6* mutants are the most well characterized. The *arc5* mutant has a reduced number of chloroplasts, all of which are arrested at a late stage of division, in mature mesophyll cells [5,6]. The *arc6* mutant exhibits a more extreme phenotype, with each mesophyll cell containing only one or two expanded chloroplasts [7]. *ARC5* encodes a cytoplasmic dynamin-related protein (DRP5B), while *ARC6* encodes an inner envelope-spanning protein that anchors the prokaryotic tubulin-like GTPase FtsZ to promote its self-assembly into a stromal division ring (FtsZ ring) [8,9]. Ferns and seed plants encode two subfamilies of FtsZ proteins, FtsZ1 and FtsZ2 [10]. FtsZ1 and FtsZ2 can form heteropolymers (protofilaments), resembling those assembled from α- and β-tubulins within microtubules, which further bundle into a contractile ring [11]. ARC6 interacts specifically with FtsZ2 [12] and could have a role in tethering the FtsZ ring to the inner envelope. ARC5/DRP5B and FtsZ form distinct concentric rings at the chloroplast division site and on the cytoplasmic and stromal surface of the envelope, respectively. Spatial coordination of those two rings across two membranes (the outer and inner envelopes) is achieved by a chain of protein interactions, namely ARC5–PDV1/PDV2 (PLASTID DIVISION1/2; bitopic outer envelope membrane proteins homologous to each other) in the cytosol [13], PDV2–ARC6 in the intermembrane space [14,15], and ARC6–FtsZ2 in the stroma [12]. In addition to these protein-protein interactions, the chloroplast phenotypes of *arc5* and *arc6* and the properties of ARC5 and ARC6 proteins provide valuable insights into the molecular components of the division machinery on both sides of the envelope, the evolutionary process by which such machinery was established, and the orderly progression of events that occurs during division: division initiation (involving ARC6), membrane constriction (FtsZ), and final separation of the daughter chloroplasts (ARC5).

Leaf epidermal cells provide another model for studying chloroplast (plastid) division. The epidermal system of *Arabidopsis* leaves comprises pavement cells (PCs), stomatal guard cells (GCs) containing immature, chlorophyll-bearing chloroplasts, and unicellular trichomes containing achlorophyllous leucoplasts, as demonstrated by microscopy analysis and explored in a recent review [16,17]. In *arc5* and *arc6*, a significant reduction in chloroplast number and a concomitant increase in chloroplast size were observed in PCs and stomatal GCs, in addition to mesophyll cells [5–7,18]. Furthermore, in *arc6*, some stomata completely lack chloroplasts and some contain one or two chloroplasts exclusively in one of the paired GCs [18]. Moreover, analysis of stroma-targeted fluorescent proteins revealed that the chloroplast-deficient GCs in *arc6* contain non-green plastids and that approximately 20% of GCs lack any type of plastid in the cotyledons of the *A*. *thaliana crumpled leaf* (*crl*) mutant [19], which displays abnormal plastid division and overall plant development [20]. These phenotypes could not be explained by the known properties of CRL protein. These observations, together with studies investigating leaf epidermal chloroplasts in the tomato *suffulta* mutant [21], epidermal plastids in the hypocotyls, petals, and stamen filaments of *arc3*, *arc5*, and *arc6* [22], and chloroplasts in the embryos, cotyledon epidermis, and leaf epidermis of *arc6* and *crl* [19], prompted us to investigate the tissue-dependent patterns of plastid (chloroplast) division.

In this context, we previously reported the plastid phenotypes in cotyledons, floral organs, and roots of the *A*. *thaliana atminE1* mutant [23], in which the expression of the gene for a prokaryote-derived, chloroplast division site-determining factor, MinE [24–26], is severely knocked down, and thus mesophyll chloroplasts appear unable to initiate the division process [27]. Whereas *atminE1* mesophyll cells uniformly contain one or a few expanded chloroplasts per cell, like those of *arc6*, the size distribution of plastids in mature root tissues (except for the columella) of *atminE1* does not significantly differ from that in the wild type (WT), indicating that MinE contributes relatively little to plastid division in roots. In addition, we recently found that *atminE1* PCs exhibit some distinct phenotypes [28]: (1) heterogeneous patterns of plastid size and shape, which vary on a cell-by-cell basis; (2) prominent induction of stromule formation and elongation, similar to that reported for several types of non-mesophyll plastids in *arc3*, *arc5*, and *arc6* [22,29]; and (3) the formation of a grape-like plastid cluster, a plastid morphology that was described for the first time. Although it remains unclear how these grape-like plastid clusters are generated in *atminE1* PCs, we hypothesized that they might actually be derived from a single plastid (or a few plastids) with many stromule-derived subcompartments (or “bulges”) [28]. These bulges might undergo fission, possibly *via* an alternative mode of plastid replication involving budding and stromule breakage [21,30], but they might fail to separate from one another for an unknown reason. Since the alternative replication mode is assumed to operate only when “conventional” plastid division dependent on the FtsZ/dynamin system is perturbed [21,31], it would be useful to examine the plastid phenotypes in the leaf epidermis of the well-studied chloroplast division mutants, *arc5* and *arc6*, in order to obtain further insight into the alternative (and perhaps non-mesophyll-specific) mode of plastid division. Therefore, in the current study, we investigated plastid morphology in the epidermis of mature leaves from *arc5* and *arc6* and compared it with that of *atminE1*.

## Materials and methods

### Plant materials and growth conditions

Seeds from *arc5* (stock number CS1633; *arc5-1* allele [5]) and *arc6* (stock number CS288; *arc6-3* allele [9]) and from *atminE1* (stock number DLFTV7T3; FLAG_056G07 T-DNA insertion line [32]) were obtained from the Arabidopsis Biological Resource Center (ABRC, Ohio State University, USA) and Institut National de la Recherché Agronomique (INRA, Versailles, France), respectively. The plastid stroma was fluorescently labeled in the living tissues of three transgenic lines in the WT background, ptA5-3 (genotype *CaMV35S* promoter::*TP*_*RBCS3A*_*-GFP*::*NOS* terminator [33]), FL6-4 (genotype *CaMV35S* promoter::*TP*_*FtsZ1-1*_*-YFP*::*NOS* terminator and *CaMV35S* promoter::*NLS*_*Cry2*_*-CFP*::*NOS* terminator [19]), and FL6-5 (genotype identical to FL6-4; this study); the abbreviations are as follows: *CaMV35S*, cauliflower mosaic virus 35S gene; TP, transit peptide; GFP, green fluorescent protein; NOS, nopaline synthase; CFP, cyan fluorescent protein; NLS, nuclear localization signal. FL6-5, a sister line to FL6-4 obtained in a previous transformation experiment [19], was used to produce *atminE1* plants homozygous for T-DNAs carrying fluorescent protein genes. Successful crossing and seed amplification of *arc5* × ptA5-3/FL6-4, *arc6* × ptA5-3, and *atminE1* × FL6-5, as well as *arc6* × FL6-4 [19], were confirmed by observing seedlings or leaves by stereofluorescence microscopy (model FLIII, Leica Microsystems, Heidelberg, Germany) and light microscopy (model IX70, Olympus, Tokyo, Japan). These stable lines were employed for subsequent microscopy analyses. Besides the above transgenic lines, *A*. *thaliana* ecotypes Columbia (Col), Landsberg *erecta* (L*er*) and Wassilewskija (Ws) were employed as controls to examine chloroplast number per GC. Plants were germinated and grown as previously described [34], except that MS medium containing 2–3% (w/v) sucrose was used in this study. All *Arabidopsis* lines exhibited healthy growth and were fertile throughout the experiments.

### Confocal laser scanning microscopy and epifluorescence microscopy

The basal parts of the leaf including the petiole were excised from seedlings with tweezers. The samples were mounted in water under glass coverslips and analyzed by confocal laser scanning microscopy (CLSM) or epifluorescence microscopy. PCs and GCs on the adaxial side of the upper petiole region were observed.

CLSM was performed using an LSM700 system (Carl Zeiss, Jena, Germany) equipped with 63× (N.A. 1.40 oil immersion) and 100× (N.A. 1.46 oil immersion) objective lenses and an Ar laser (488 nm) as an excitation source. Maximum intensity projection images (512 × 512 pixels) were taken with LSM700 software. For segmentation and reconstruction of fluorescent protein-labeled plastid structures, individual CLSM images were automatically segmented using the ImageJ plug-in packages “LPixel ImageJ plugins”. Noise was removed using the denoise mode “gradInvWeightedOp” with the “denoiseFilters__” of Lpx Filter2d. The LPixel ImageJ plugins are available from https://lpixel.net/en/services/lpixel-imagej-plugins. Three-dimensional reconstruction movies were generated using the ImageJ plug-in “Volume Viewer”. Epifluorescence microscopy was performed using an inverted microscope (model IX71, Olympus, Tokyo, Japan) equipped with 60× (N.A. 1.20 water immersion) and 40× (N.A. 0.95 dry) objective lenses and a mercury lamp (model USH-1030L, Ushio, Tokyo, Japan) as an excitation source. Fluorescent signals from GFP, YFP, and chlorophyll were obtained using optical filters and a CMOS digital camera (model ORCA-flash2.8, Hamamatsu Photonics, Hamamatsu, Japan) as previously described [28]. In both CLSM and epifluorescence microscopy, bright-field images were taken with differential interference contrast (DIC) optics.

### Measurement of stomatal GCs and plastids in leaves

For quantitative analyses of GCs and their plastids (chloroplasts), the adaxial sides of the upper petiole and blade/petiole junction regions from fully expanded leaves were used. All experiments were performed using samples under the same conditions: the basal parts of third-fourth leaves containing petioles were excised from 4-week-old WT, *arc5*, *arc6*, and *atminE1* seedlings, which were grown on 2% (w/v) sucrose-containing MS plates under daily irradiation from 5:00 to 21:00. Measurements of chloroplast (body) length were performed using leaves sampled from 14:00 to 17:00 *via* epifluorescence microscopy as described above. The frequency of GCs with giant chloroplasts, with normal-sized chloroplasts, or lacking chloroplasts was also counted. GC chloroplasts were identified using DIC and epifluorescence microscopy, and their sizes were defined as follows: giant chloroplasts, >6 μm; normal-sized chloroplasts, 2–6 μm; and mini-chloroplasts, <2 μm.

## Results

### Plastid morphology in leaf PCs of *arc5* and *arc6*

To investigate the morphology of leaf epidermal plastids in the *Arabidopsis arc5* and *arc6* mutants, we introduced expression cassettes encoding stroma-targeted YFP or GFP under the control of the *CaMV35S* promoter into both mutants *via* crossing (for details, see the Materials and Methods). All offspring exhibited healthy growth and development under laboratory conditions and gave consistent results with regard to plastid morphology (data not shown). Unless otherwise specified, plastid image data were obtained from WT, *arc5*, and *arc6* plants in which stroma were labeled with YFP and detected by CLSM.

**Fig 1:**
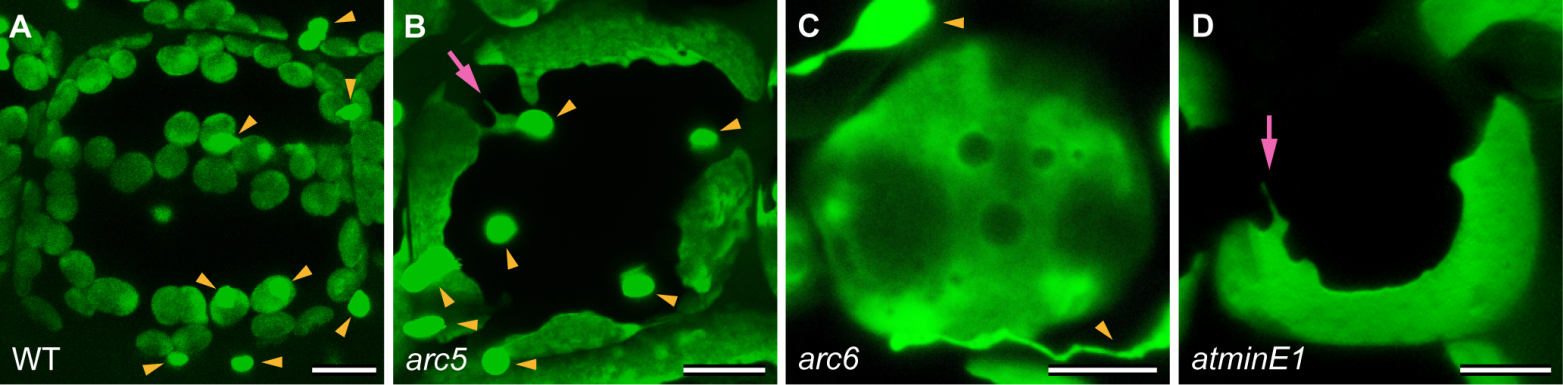
Plastid phenotypes in leaf mesophyll cells of *Arabidopsis* WT, *arc5*, *arc6*, and *atminE1*. (A–D) Images of plastid-targeted YFP in the third-fourth leaf petioles of 4-week-old WT (A), *arc5* (B), *arc6* (C), and *atminE1* (D) seedlings. CLSM images of maximal intensity projection are shown. Arrowheads and arrows indicate epidermal PC plastids and stromules from enlarged plastids, respectively. Bar = 10 μm.

In the cortex cells of leaf petioles from *arc5* and *arc6*, plastids were greatly enlarged compared with the WT (Fig 1). In particular, the cortex cells of *arc6* contained only one or a few extremely large plastids resembling those of *atminE1* [27], implying severe inhibition of cortex plastid division in both mutants. The phenotypes of plastids in *arc5* and *arc6* leaf petioles were essentially the same as those in leaf blade mesophyll cells observed with DIC optics [5,6] and with GFP fused to a transit peptide [19,22]. Therefore, the introduction of the stroma-targeted *YFP* or *GFP* gene did not appear to affect the phenotypes of either *arc* mutant. In the current study, we evaluated plastid morphology in the epidermis of leaf petioles from the above transgenic lines, focusing on four aspects of plastid morphology.

#### Giant plastid formation

**Fig 2:**
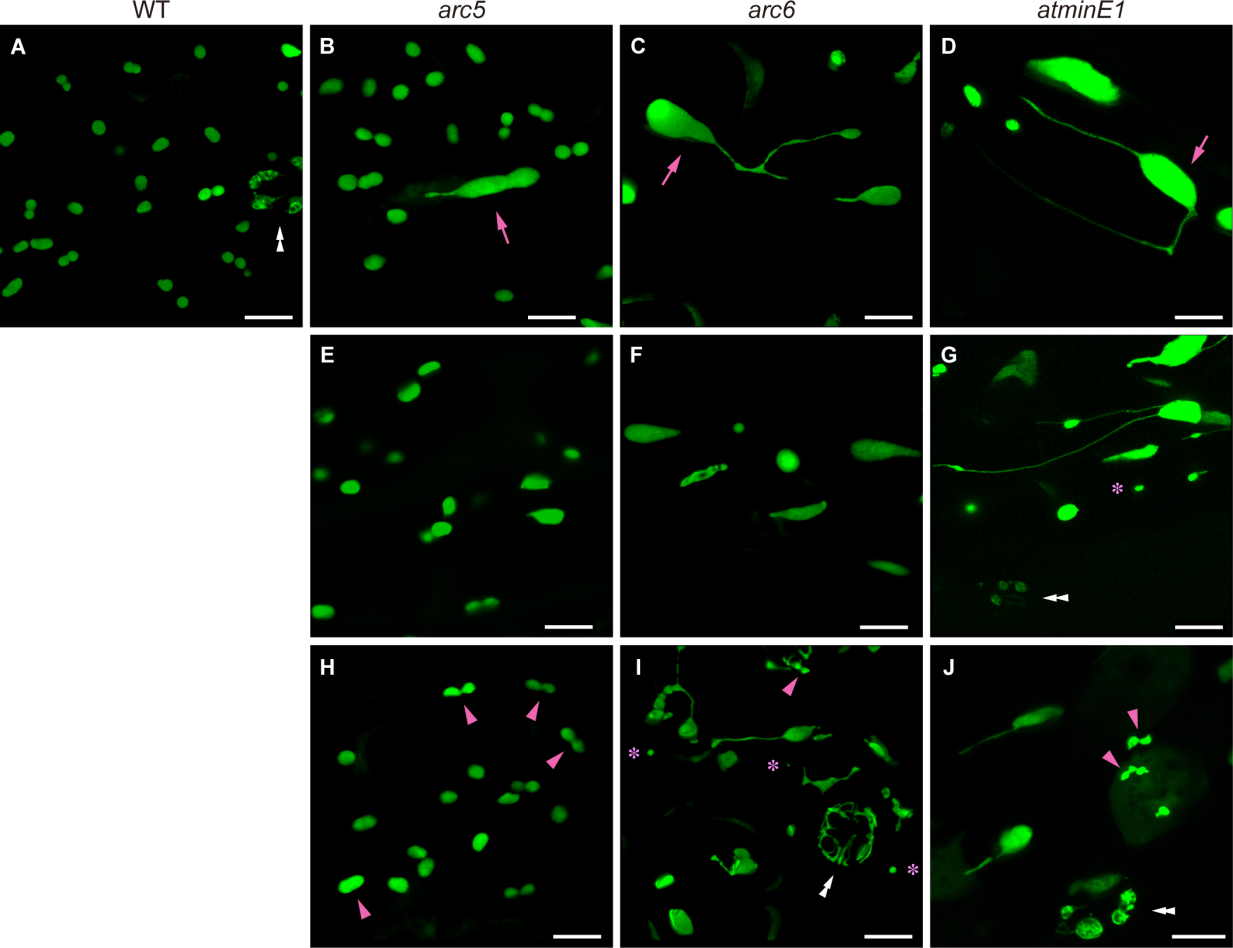
Plastid phenotypes in leaf PCs of *Arabidopsis* WT, *arc5*, *arc6*, and *atminE1*. (A–J) Images of plastid-targeted YFP in the third-fourth leaf petioles of 4-week-old WT (A), *arc5* (B, E, H), *arc6* (C, F, I), and *atminE1* (D, G, J) seedlings. CLSM images of maximal intensity projection are shown. Giant plastids (arrows), putative dividing plastids (arrowheads), stomata (double arrowheads), and mini-plastids (asterisks) are indicated. Bar = 10 μm.

Enlarged plastid size is a primary index used to assess defects in plastid division [35]. Using CLSM, we found that WT plastids in *Arabidopsis* leaf petiole PCs were round to ellipsoidal, or occasionally dumbbell-shaped, and were approximately 2–5 μm long (Fig 2A). This result is in agreement with previous observations of PCs in leaf blades and petioles obtained by fluorescence, confocal, and electron microscopy [6,16,28,34,36]. Based on this information, we defined “giant plastids” as enlarged plastid bodies >6 μm long. Whereas we did not detect giant plastids in the WT (Fig 2A), we observed them in *arc5*, although at very low frequency (Fig 2B). On the other hand, we detected giant plastids in most PCs of *arc6* (Fig 2C). In both mutants, giant plastids assumed a non-fixed shape, developed extended stromules, and did not exhibit obvious constrictions, although an array of giant plastid bodies inter-connected by this tubular structure, which locally resembled stromules, were rarely observed in *arc6*. Nevertheless, the mutant plastid bodies appeared mostly spherical, a modest phenotype relative to mesophyll plastids, which exhibited extensive, irregular expansion in three dimensions [7,18]. We never detected PCs without plastids in either mutant during our microscopic observations.

These results strongly suggest that ARC5 and ARC6 are involved in epidermal plastid morphogenesis in leaf PCs. The rare but actual occurrence of giant plastids in *arc5* PCs was never before observed in the shoot epidermis of this mutant. Its frequent occurrence in *arc6* PCs is comparable to that in other epidermal and leaf mesophyll cells of the mutant [7,18,22,29]. These results suggest that, in leaf PCs, ARC6 is critical for normal plastid division, while ARC5 is less important in these cells than in mesophyll cells or ARC6.

#### Size homogeneity/heterogeneity of plastids

Next, we focused on the plastid populations in PCs. As described above, the WT cells showed slight variation in plastid size (Fig 2A). On the other hand, *arc5* displayed a moderate level of plastid size polymorphism, ranging from a slightly reduced size to a relatively large size, although many *arc5* plastids were slightly larger than those in the WT (Fig 2E). By contrast, *arc6* exhibited marked polymorphism in plastid size, ranging from mini-plastids (defined as plastids <2 μm in length) to giant plastids (Fig 2F). In both mutants, plastids of different sizes coexisted within the respective PCs, although their distribution patterns varied between PCs.

The size heterogeneity of *arc6* PC plastids was more or less associated with their varied shapes. A majority of mini-plastids were vesicular (Fig 2I), although some were elongated (data not shown). Extensive structural variations in *arc6* plastids were observed in the larger-sized plastid group. These plastids were polymorphic in shape, including spherical, elongated, wavy, multi-lobed, or amoeboid shapes (Fig 2I). Also, *arc6* PCs contained poorly developed plastids that were capable of forming isthmi (Fig 2I), whereas WT and *arc5* cells contained dumbbell-shaped plastids that were likely undergoing symmetric binary fission (Fig 2A and 2H).

Our findings about plastid size distribution in the leaf petiole PCs of *arc5* and *arc6* complement those obtained in leaf blades [5–7], and reveal an unexpectedly large amount of size heterogeneity of PC plastids in *arc5* and *arc6*. In addition, the phenotypes of *arc5* and *arc6* leaf PCs strongly resembled those of stamen filaments, among previously examined tissues of the mutants [22].

#### Stromule development

Stromules are subplastidic structures that exhibit a tubular form and extend into the cytoplasm. These structures comprise double-envelope membranes and the stroma, and can emanate from, or retract back to, the plastid surface on the order of seconds. Stromule formation is generally active in non-photosynthetic tissue cells, and in shoots, stromules preferentially occur in the PCs of hypocotyls and leaves [37–39].

In the WT, stromules were detected at low frequency (not shown). In *arc5*, the frequency of stromule formation was similar to that in the WT, except for the prominent stromule formation from giant plastids (Fig 2H). In both lines, we often failed to detect plastids with stromules in certain PCs, whereas they were readily detected in other cases. Nevertheless, *arc5* cells occasionally contained long, stable stromules, which were never detected in the WT. In *arc6*, stromules formed frequently and grew extensively (Fig 2I). Some of these stromules assumed a “beads-on-a-string” structure, which has been reported for several types of non-green plastids [40,41], and others formed intricate networks (S1 Fig). These plastid body and stromule configurations in *arc6* resembled those reported in the epidermal tissues of this mutant other than leaves [22] and in those of *atminE1* [28] (see below). Furthermore, in all plants, the stromules exhibited highly dynamic transformation activities, such as emergence, extension, retraction, and branching, during our microscopic observations (data not shown).

#### Grape-like plastid clusters

**Fig 3:**
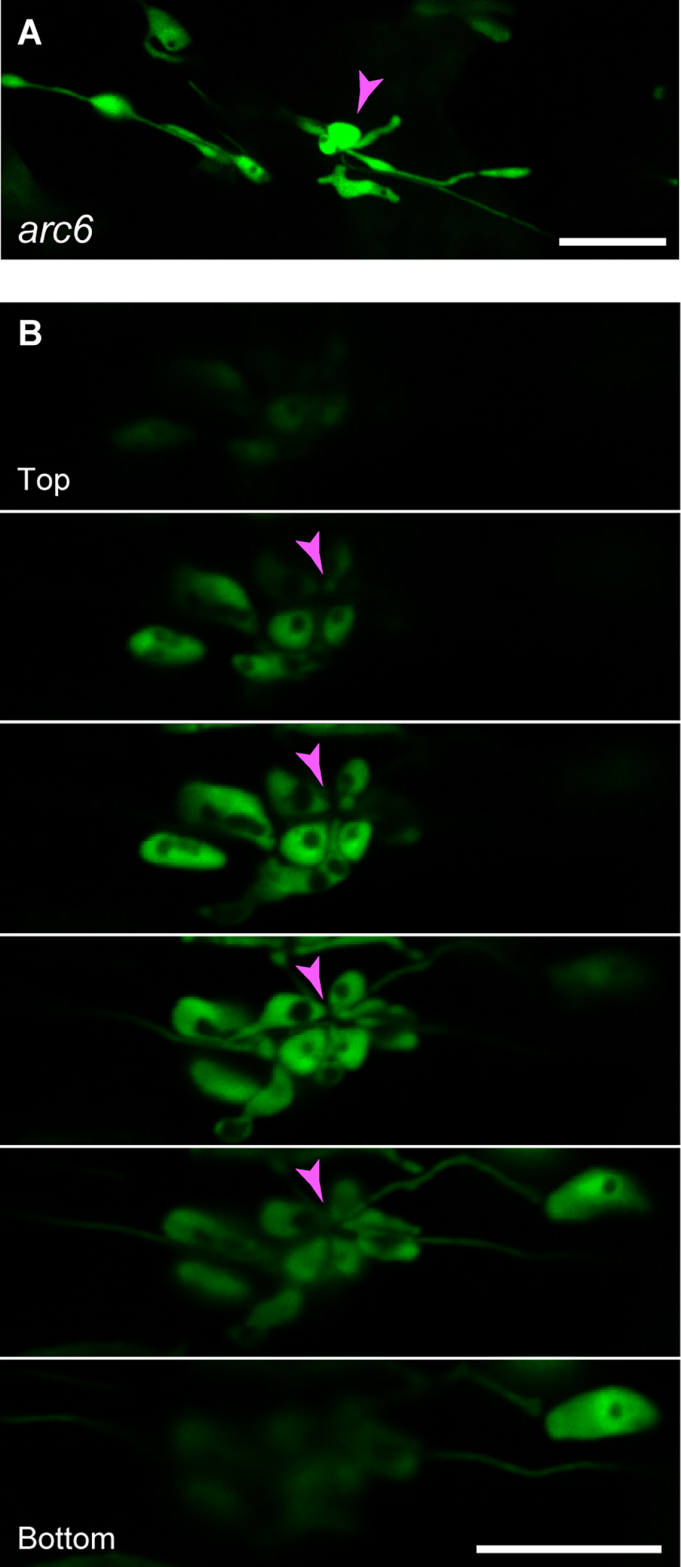
Grape-like plastid clusters in leaf PCs of *arc6*. Images of plastid-targeted YFP in the third-fourth leaf petioles of 4-week-old seedlings from *arc6* are shown. (A) Formation of plastid bulges. (B) Serial optical sections of grape-like plastid clusters. Arrowheads indicate the activated regions of plastid bulges. Bar = 10 μm.

We previously reported a novel plastid morphological phenotype: the grape-like clustering of leaf PC plastids in *atminE1* [28]. To date, this phenotype has been reported only for *atminE1*. In the present study, we investigated whether the PCs in *arc5* and *arc6* also possess grape-like plastid clusters, finding that this type of structure was present in *arc6*, but not in *arc5* (Fig 3). The grape-like plastid clusters in *arc6* (Fig 3A) were structurally similar to those in *atminE1* [28]. Taking advantage of CLSM, we obtained optical sectioning images of this plastid structure (Fig 3B). In the serial *z*-stack images, we did not observe any node-like structures at the center (or the bulge-gathering site) of the plastid clusters. Therefore, the absence of such a “central node” in the grape-like plastid clusters was observed in both *arc6* (this study) and *atminE1* [28].

In characterizing *arc5* and *arc6*, we employed the *atminE1* mutant, a resource for studying tissue-dependent plastid morphogenesis, as a reference [23,27,28,34,42]. In all aspects of plastid morphology examined in this study, *i*.*e*., giant plastid formation (Fig 2D), the size distribution of plastids (Fig 2D and 2G), stromule formation (Fig 2D, 2G and 2I), and grape-like plastid clustering (data not shown), *atminE1* strongly resembled *arc6*. This result is in accordance with the plastid morphology observed in leaf mesophyll cells, cotyledons, petals, stamen filaments, stem endodermis, and roots [7,18,19,22,23,27,42–44]. In addition, FtsZ proteins (FtsZ1 and FtsZ2) exhibit similar localization patterns in the giant chloroplasts of *arc6* and *atminE1* (and *arc12*, another mutant allele of *AtMinE1* [45,46]) [9,27,34,46–49]. Thus, ARC6 and AtMinE1 may play equally important roles in plastid morphogenesis in leaf PCs as they do in mesophyll cells. By contrast, we failed to detect obvious similarity in plastid phenotypes in PCs (or in cells in other tissues) between *atminE1* and *arc5*, except at the individual plastid level.

### Plastid morphology in leaf stomatal GCs of *arc5* and *arc6*

Extending the analysis of epidermal plastids in leaf PCs, we examined those in stomatal GCs. GCs are specialized epidermal cells that control gas flux between the plant body and the atmosphere [50]. They contain photosynthetic plastids (chloroplasts), whose morphogenesis is an important subject of study; the generation of chloroplast-deficient GCs in *arc6* leaves was the first experimental demonstration that the inhibition of correct chloroplast division affects the photosynthetic ability of plant cells [18].

In general, in all WT and mutant lines (*arc5*, *arc6*, and *atminE1*) examined, we failed to detect GCs devoid of plastids. Meanwhile, we observed previously unreported plastid phenotypes in the GCs of the above mutants; the morphological patterns of the GC plastids strongly varied on a cell-by-cell basis, and thus it was difficult to define a “typical” plastid phenotype for each mutant. Instead, we defined these phenotypes in terms of multiple morphological patterns as follows.

#### WT

**Fig 4:**
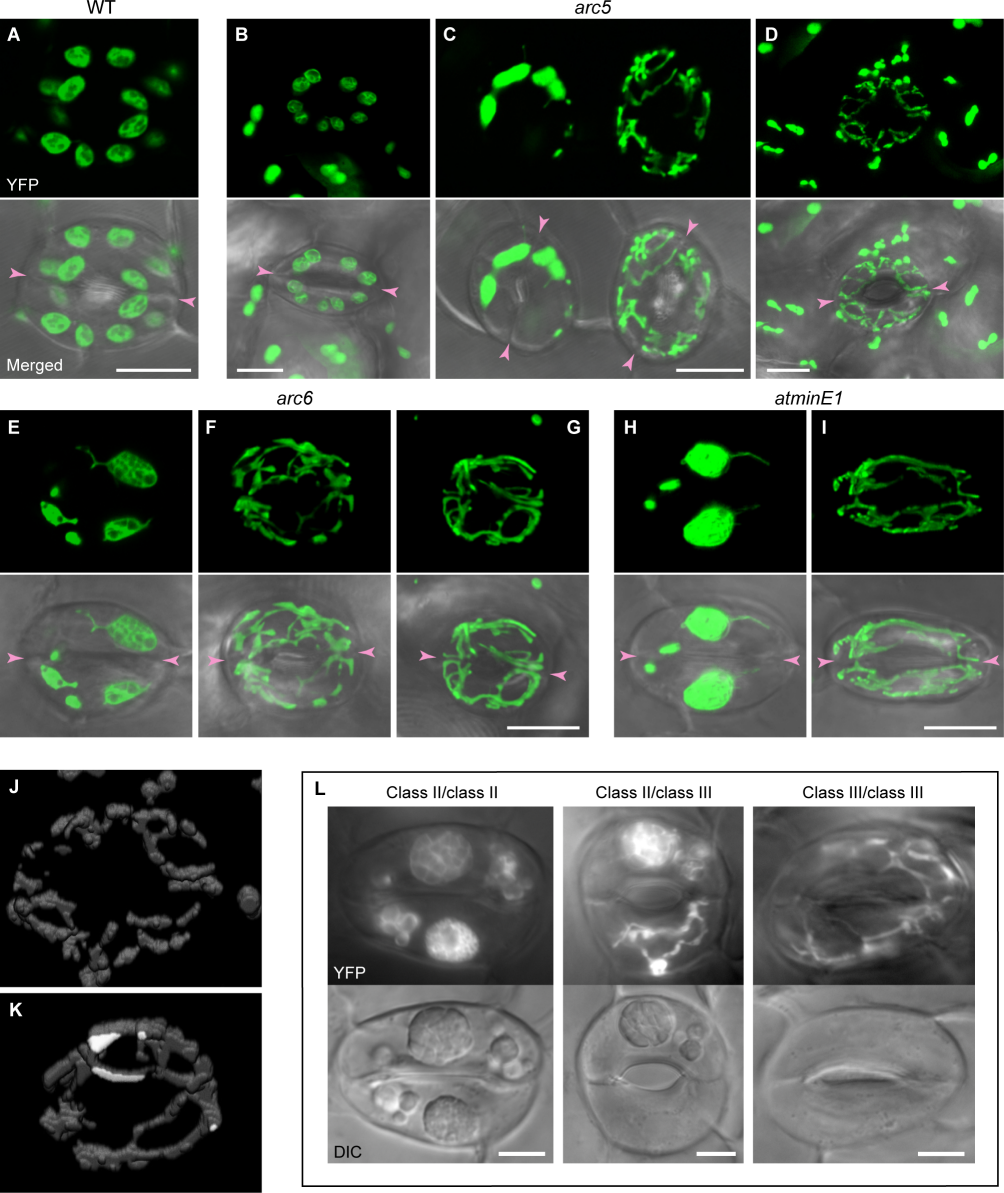
Plastid phenotypes in leaf stomatal GCs in *Arabidopsis* WT, *arc5*, *arc6*, and *atminE1* plants. (A–I) Images of plastid-targeted YFP in the third-fourth leaf petioles of 4-week-old seedlings from the WT (A), *arc5* (B–D), *arc6* (E–G), and *atminE1* (H, I). CLSM images of maximal intensity projection (top panels) and merged with DIC (bottom panels) are shown. (J, K) Images of YFP-labeled plastids in GCs reconstructed from a series of optical sections generated by CLSM, taken at 0.3 or 0.6 μm intervals. The GC pairs in panels (J) and (K) are identical to those in panels (D) and (G), respectively. (L) Epifluorescence microscopy images of GCs in the third-fourth leaf petioles of 4-week-old seedlings from *arc6*. Images of YFP (top panels) and DIC (bottom panels) are shown. Bar = 10 μm.

GC plastids in the rosette leaves and cotyledons of WT *Arabidopsis* plants were described in earlier studies [5,7,16,18,19,51]. In accordance with these reports, we found that leaf petiole GCs in the WT contained round or ellipsoidal plastids, which were generally equal in size (average length of ~4 μm) and uniformly dispersed in the cytoplasm (Fig 4A).

#### arc5

In *arc5*, the morphological patterns of plastids in GCs were roughly grouped into three classes: homogeneous population of normally sized and shaped plastids like the WT (class I; Fig 4A and 4B); abnormal plastid population, often represented by the presence of giant or minute plastids (class II; Fig 4C, in the left GC pair); and populations of numerous minute plastids, which were colorless (chlorophyll-less), as confirmed by bright-field microscopy (class III; Fig 4C, in the right GC pair; Fig 4D and S2 Fig). Class III GCs usually contained more plastids per cell than WT GCs, although individual class III plastids were more difficult to discriminate (S2 Fig). The occurrence of class III GCs was occasionally accompanied by surrounding PCs with poorly developed plastids (compare Fig 4D with Fig 4B).

#### arc6

In *arc6*, GCs containing WT-like normal plastids only (*i*.*e*., class I) were absent, while class II and III GCs were observed (Fig 4E and 4F and S3 Fig). Importantly, in contrast to the previous observation that most GCs of *arc6* leaves and cotyledons contain a single enlarged plastid [18,19], a majority of class II GCs in our samples contained several plastids of various sizes. In addition, whereas class III-like GCs in *arc6* were previously reported [19], we found that, among class III GCs, the plastid number per cell varied more widely than previously reported, ranging from a few to many, highly proliferated plastids. Furthermore, in some *arc6* GCs, we observed web-like structures consisting of chlorophyll-less plastids (class IV) (Fig 4G and S3 Fig). By definition, class IV plastids had a more severe phenotype than class III plastids, but we were often unable to discriminate between these classes. Remarkably, in class IV GCs, plastids appeared to lose the recognizable “main body” and become entirely tubulate.

#### atminE1

**Fig 5:**
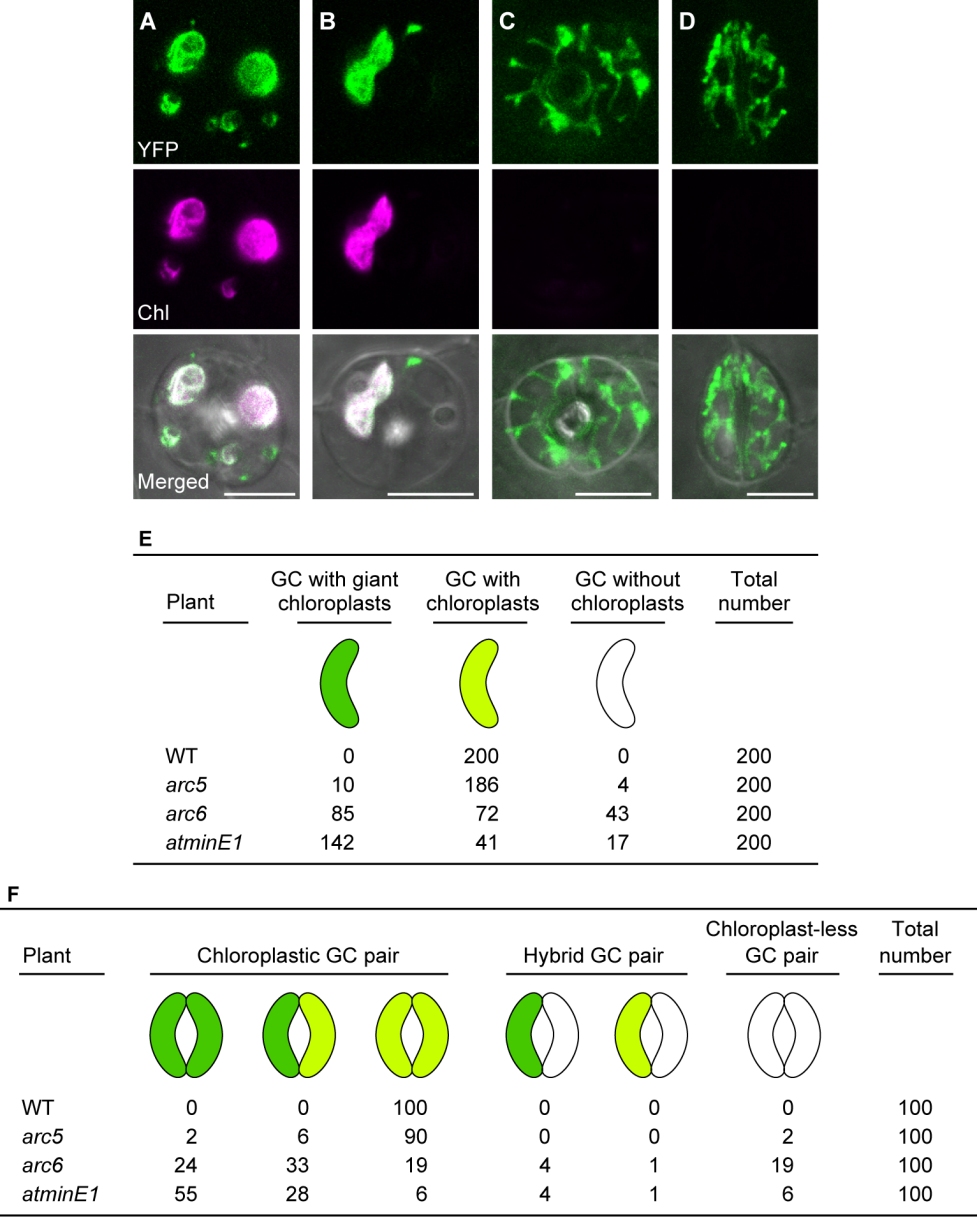
Classification of leaf stomatal GCs in *Arabidopsis* WT, *arc5*, *arc6*, and *atminE1*. (A–D) Images of stomatal GCs in the third-fourth leaf petioles of 4-week-old *atminE1* seedlings. CLSM images of maximal intensity projection for plastid-targeted YFP (top panels) and chlorophyll autofluorescence (Chl; middle panels) and merged images with DIC (bottom panels) are shown. Bar = 10 μm. (E, F) Frequency of GCs with giant or normal-sized chloroplasts or lacking chloroplasts. In (E, F), the frequency of GCs in individuals (E) or paired cells (F) was counted in arbitrarily selected cells in the WT, *arc5*, *arc6*, and *atminE1*.

The plastid morphological patterns in the *atminE1* GCs resembled those in *arc6*; whereas WT-like plastids (class I) were never observed, class II, III, and IV GCs were observed (Fig 4H and 4I, Fig 5A–5D, and S4 Fig). This result supports the observation that the plastid phenotype of *atminE1* is similar to that of *arc6*, in GCs as well as in PCs.

To confirm the unusual plastid morphologies in mutant GCs, we acquired three-dimensional images of class III plastids (Fig 4J and S1 Mov, from an *arc5* sample) and class IV plastids (Fig 4K and S2 Mov, from an *atminE1* sample), which clearly illustrate the structure and distribution of mini-plastids (class III) and web-like plastids (class IV) in GCs. These plastids were dispersed throughout the GC cytoplasm at high density. Further fluorescence microscopy analysis also supported that plastid imaging was not seriously affected by starch granules in these cases (Fig 4L). To our knowledge, such distribution patterns of plastids have never been described in epidermal tissues of *Arabidopsis* shoots, and hence they might represent novel, extreme plastid phenotypes caused by mutations in plastid-division genes.

### Quantitative evaluation of plastid morphology, pigmentation, and partitioning in leaf stomatal GCs in *arc5* and *arc6*

We then observed chlorophyll autofluorescence and stromal YFP fluorescence simultaneously in GCs, as exemplified in *atminE1* (Fig 5A–5D). The giant plastids (>6 μm in length) observed in class II GCs always emitted intense chlorophyll autofluorescence (Fig 5A and 5B), confirming that they were genuine chloroplasts. Among class II non-giant plastids in the same GCs, some were chloroplasts and others were chlorophyll-less plastids (Fig 5A and 5B). By contrast, all plastids in class III and IV GCs were chlorophyll-less (Fig 5C and 5D). We believe that such chlorophyll-less plastids bore no or only trace amounts of chlorophyll, as in the case of plastids in *albino* plants. These results reveal the association of the morphological patterns of GC plastids (*i*.*e*., classes I–IV) with the status of plastid differentiation (greening).

**Fig 6:**
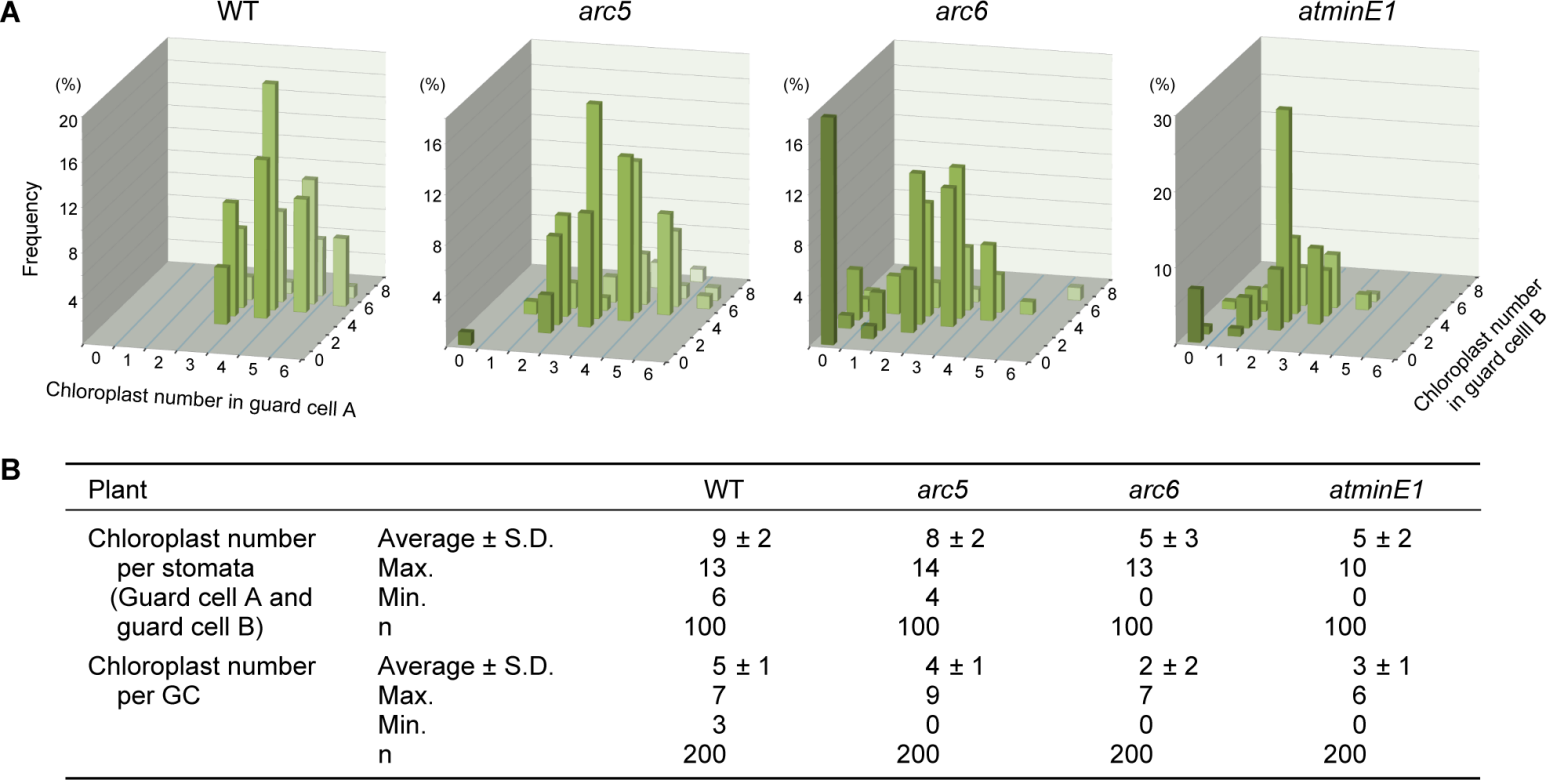
Distribution of chloroplasts in leaf stomatal GCs of *Arabidopsis* WT, *arc5*, *arc6*, and *atminE1* plants. (A, B) Measurements of chloroplast number in arbitrarily selected pairs of GCs (‘cell A’ and ‘cell B’) are shown in graph (A) and table (B) form.

Based on the above observations, we classified GCs into three types: GCs containing giant chloroplasts, which covers part of class II (type 1); GCs containing normal-sized chloroplasts but no giant chloroplasts, which covers class I and the remainder of class II (type 2); and GCs not containing chloroplasts, which covers classes III and IV (type 3). Note that we never detected GCs containing only mini-chloroplasts in this study. We counted the occurrence of each GC type (Fig 5E and 5F) and the chloroplast number per GC (Fig 6) and measured the sizes of GC chloroplasts (Table 1) in the WT, *arc5*, *arc6*, and *atminE1*. Since a pair of GCs is terminally produced through symmetric division of the guard mother cell (GMC), we counted these chloroplasts in both single GCs and GC pairs to gain insight into plastid partitioning upon cell division. Fig 5B shows an extreme case of asymmetric plastid partitioning between paired GCs: one with a giant chloroplast and the other with a minute, non-colored plastid. Such a situation was never reported for mesophyll cells, and hence might be unique to GC pairs.

**Table 1:** Chloroplast length in leaf stomatal GCs of the WT, *arc5*, *arc6*, and *atminE1*^1^.

Plant	WT	*arc5*	*arc6*	*atminE1*
Frequency of chloroplasts				
Giant chloroplast	1 (0.5%)	7 (3.5%)	64 (32.0%)	55 (27.5%)
Normal-sized chloroplast	199 (99.5%)	191 (95.5%)	102 (51.0%)	119 (59.5%)
Mini-chloroplast	0 (0.0%)	2 (1.0%)	35 (17.0%)	26 (13.0%)
Chloroplast length (μm)				
Average ± S.D.	4.3 ± 0.8	4.8 ± 1.0	5.2 ± 3.1	5.3 ± 2.7
Max.	6.8	10.2	14.1	14.5
Min.	2.9	1.5	1.0	1.5
Total number of chloroplasts examined	200	200	200	200

^1^ For experimental details, see the Materials and Methods.

#### WT

Virtually all WT GCs fell into type 2 (Fig 5E and 5F and Table 1) and contained 5 ± 1 chloroplasts (max. 7; min. 3) per cell (Fig 6B). The vast majority of these chloroplasts were approximately 3–5 μm in length (Table 1). At the GC-pair level, the most frequent pattern of plastid partitioning was ‘cell A’:‘cell B’ = 4:5 (Fig 6A). Under the same experimental condition, non-transgenic plants of three *A*. *thaliana* ecotypes, Col, L*er* and Ws, exhibited similar results to the WT, with respect to chloroplast number per stomata (for Col, 10 ± 1 on average, max. 15, min. 7 [n = 100]; for L*er*, 11 ± 2 on average, max. 16, min. 8 [n = 100]; for Ws, 10 ± 2 on average, max. 16, min. 7 [n = 100]) and GC (for Col, 5 ± 1 on average, max. 8, min. 3 [n = 200]; for L*er*, 6 ± 1 on average, max. 8, min. 3 [n = 200]; for Ws, 5 ± 1 on average, max. 8, min. 3 [n = 200]).

#### arc5

Most *arc5* GCs fell into type 2, whereas type 1 and 3 GCs were also rarely observed (Fig 5E and Table 1). The GCs contained 4 ± 1 chloroplasts per cell (Fig 6B). On average, these chloroplasts were only slightly larger than those of the WT, and a large portion of *arc5* chloroplasts were comparable to those of the WT in terms of size (Table 1). Nevertheless, the size range for *arc5* chloroplasts (1.5–10.2 μm) was markedly higher than that in the WT (2.9–6.8 μm) (Table 1). Interestingly, at the GC-pair level, type 3 (chloroplast-less) GCs formed pairs with other type 3 GCs (Fig 5F).

#### arc6

A considerable number of *arc6* GCs fell into type 1 and 3 (43% and 22%, respectively), in addition to type 2 (36%) (Fig 5E and Table 1). The GCs contained 2 ± 2 chloroplasts per cell (Fig 6B) that were larger than those of the WT, on average (Table 1). The chloroplasts ranged in size from 1.0 to 14.1 μm, although it is possible that tiny chloroplasts below the detection limit of current microscopy might also exist. This size range is even broader than that detected in *arc5* (Table 1). At the GC-pair level, type 3 GCs had a tendency to pair with other type 3 GCs and to avoid “hybrid” pairing with types 1 and 2 (Figs 5F and 6A). This pattern of pairing is similar to that in *arc5* (Fig 5F). Our results also indicate that GCs in *arc5* and *arc6* possess more chloroplasts per cell than previously described [7,19].

#### atminE1

The GCs from *atminE1* fell into types 1 and 3 (at a level of 71% and 9%, respectively), in addition to type 2 (21%) (Fig 5E and Table 1). These GCs possessed 3 ± 1 chloroplasts per cell (Fig 6B), which were larger, on average, than those of the WT (Table 1). The chloroplasts ranged from 1.5 to 14.5 μm in size, which is similar to that in *arc6* (Table 1). These quantitative data essentially support the similar morphological patterns of GC plastids detected in *arc6* and *atminE1*, as shown in Fig 4. However, there were some differences between these mutants. For example, at the single-GC level, type 1 GCs were 1.7-fold more frequent in *atminE1* than in *arc6*, whereas type 3 was 2.5-fold less frequent in *atminE1* than in *arc6* (Fig 5E). When examined at the GC-pair level, this tendency was further reinforced; type 1/type 1 pairs were 2.3-fold more frequent in *atminE1* than in *arc6*, whereas type 3/type 3 pairs were 3.2-fold less frequent in *atminE1* than in *arc6* (Fig 5F).

To numerically evaluate the pairing preferences of GCs in the mutants, we conducted a simulation of random pairing among 200 GCs (resulting in 100 GC pairs), among which the number of each GC type was assumed to be that detected in the mutants by counting, as indicated in Fig 5E. A comparison of actual and simulated counts of each GC-pair type in *arc6* revealed a strong preference for type 3/type 3 pairings and an avoidance of hybrid pairings (type 1/type 3 and type 2/type 3) (S5 Fig; see also Figs 5F and 6A). In *atminE1*, essentially the same tendency of GC pairing was observed, although the difference between the actual and simulated counts was more modest than that observed for *arc6*. Likewise, as for *arc5*, we detected a preference of type 3/type 3 pairing and an avoidance of hybrid pairings. The difference between the actual and simulated counts for *arc5* was generally small compared with those for *arc6* and *atminE1*, probably due to the low actual numbers of type 1 (10/200) and type 3 (4/200) GCs in *arc5* (Fig 5E).

## Discussion

### Exploring differences between PC plastid division and mesophyll chloroplast division

In this study, we investigated the plastid phenotypes in leaf epidermal cells of the *arc5* and *arc6* mutants, which showed distinct morphological differences compared with the known chloroplast phenotypes reported for mesophyll cells of the corresponding mutants [5–7]. In *arc5* mesophyll cells, the chloroplasts successfully initiate the division process but are unable to complete the daughter-chloroplast separation. As a result, all *arc5* chloroplasts maintain a central constriction [6]. Since the *arc5* mesophyll chloroplasts continue to grow without division, they are 6-fold larger than the WT at maturity. By contrast, in the present study, we found that the proportion of PC plastids with an obvious central constriction in *arc5* was not clearly different from that in the WT and that their average size was only slightly larger than that in the WT (Fig 2). Therefore, despite the known phenotype of the *arc5* mesophyll chloroplasts, a large portion of *arc5* PC plastids are capable of completing their division process. The *ARC5* gene product, DRP5B, assembles into a constriction ring at the central division site on the cytosolic surface of the chloroplast, and its *Cyanidioschyzon* homolog generates the force needed for constriction *via* the chloroplast division machinery [8] (for a review, see [52]). Our results raise new possibilities for the role of DRP5B in plastid division. The first possibility is that DRP5B is only required for the fission of well-expanded or expanding plastids, such as those observed in mesophyll cells, but it is dispensable for poorly sized plastids, such as those observed in PCs. The second possibility (which is not exclusive of the first) is that PCs contain a plastid division machinery that is distinct from that of mesophyll chloroplasts and does not involve DRP5B. The possibility that DRP5B is dispensable for plastid division in certain types (or developmental stages) of cells is supported by an earlier report showing that the expression of DRP5B was too low to detect plastidial DRP5B rings in shoot apical meristems and young emerging leaves of *Arabidopsis* [53]. The possibility that dumbbell-shaped PC plastids of *arc5* contain a normal FtsZ ring at the mid-plastid constriction should be addressed in a future study. This issue is intriguing because the division-arrested mesophyll chloroplasts of *arc5* possess multiple, uncondensed FtsZ rings (or spirals) at or near the constriction site [13,54].

In the mesophyll cells of both *arc6* and *atminE1* (and another allele, *arc12*), chloroplasts are unable to initiate the division process but instead continue to expand, resulting in the uniform occurrence of mesophyll cells containing one or a few, extremely large chloroplasts [7,27,30,46], indicating that ARC6 and MinE are critical initiation factors in the conventional chloroplast division system. On the other hand, PC plastids in *arc6* and *atminE1* assume various morphological features in addition to the formation of giant plastids: size heterogeneity, extensive stromule formation, and the formation of putative division constrictions, although only in small plastids (Fig 2). ARC6 spans the inner envelope and promotes FtsZ ring formation in mesophyll chloroplasts, thereby promoting their division [9], whereas MinE (encoded by *AtMinE1* in *A*. *thaliana*) is a stromal protein that regulates FtsZ ring positioning in mesophyll chloroplasts and hence assures their symmetric division [27,34]. The distinct roles of ARC6 and MinE in chloroplast division machinery suggest that the aberrant morphologies of epidermal plastids in *arc6* and *atminE1* cannot be ascribed to a defect in the specific functions of ARC6 or MinE, but rather, they might be due to a common failure to complete an upstream event in the plastid division process (for instance, assembly of the contractile FtsZ ring).

### Plastid clustering in *arc6* leaf PCs

The finding that grape-like plastid clusters occur in *arc6* (Fig 3) and *atminE1* [28] provides important insights into the defective control of plastid division in PCs. In a previous study of *atminE1* PCs [28], we proposed that such plastid clusters occurred *via* the formation of stroma-containing “bulges”, derived from plastid bodies, stromules, or other types of plastid subcompartments, but failed to separate from one another. Within the grape-like plastid clusters in *atminE1* PCs, FtsZ1 rings were detected in the narrow regions of bulges, but not at their “basal” regions, from which the bulges appeared to arise [28]. This finding, and the absence of node-like structures connecting bulges at the center of the grape-like cluster in *arc6* (Fig 3B) and *atminE1* [28], support the hypothesis that the cluster is derived from one or a few plastids containing many bulges, at least some of which undergo fission to produce discrete plastidic entities but are incapable of detaching themselves from the site of membrane fission, and which may generally be induced by serious inhibition of PC plastid division. The process by which plastid bulges are formed, maintained, and possibly separated in *arc6* and *atminE1* PCs should be investigated in more detail to expand our knowledge of the non-mesophyll mode of plastid division.

### Plastid biogenesis during stomatal GC differentiation in the leaf epidermis of chloroplast division mutants

Stomatal GCs lacking chloroplasts, but containing chlorophyll-deficient plastids, have been observed in the tomato *suffulta* mutant [21] and *Arabidopsis arc6* and *crl* mutants [19], leading to a model in which such non-green plastids in GCs arise by budding and fragmentation of giant chloroplasts (model A). This model was originally proposed for plastid replication in the pericarp cells of ripening tomato fruits [3,21,30,55]. In a previous study based on the observation of PC chloroplasts/plastids in the *Arabidopsis atminE1* mutant, we proposed an alternative, but not mutually exclusive, model in which giant chloroplasts give rise to daughter chloroplasts or chlorophyll-less plastids through the formation of intermediate “bulge” structures (model B) [28]. We also hypothesized that these normal- or mini-sized daughter chloroplasts/plastids are capable of replicating with the aid of FtsZ1 division ring formation (model C) [28]. Conceivably, the morphological patterns of the leaf epidermal plastids in *arc5*, *arc6*, and *atminE1* observed in the present study might result from a combination of these three models. In particular, it would be intriguing to investigate whether and how the formation of class III and IV GCs in *arc6* and *atminE1* (Figs 4 and 5 and S2 and S3 Figs) can be explained by these models.

The general trend that a type 3 GC pairs with another type 3 GC, as observed in *arc6* and *atminE1* (Figs 5F and 6A), suggests that a portion of GMCs exclusively contain chlorophyll-less plastids. As represented by the class IV pattern, plastid biogenesis in GCs is a unique, markedly different process from that of the surrounding PCs, even though both GCs and PCs are present in the same epidermal tissue. Thus, in the future, it would be interesting to examine plastid biogenesis in GMCs to explore how the switching of the plastid biogenesis mode occurs.

### Insights from the varied plastid morphologies in the epidermal cells of chloroplast division mutants

Variability in chloroplast size in a mesophyll cell is an important hallmark of “division site placement” mutants, whose responsible loci encode division site positioning factors [56–62]. The current results indicate that size heterogeneity in plastids can occur in PCs and GCs of *arc5* and *arc6* (Figs 2 and 4), both with responsible loci that encode the key components of the division ring complex in mesophyll chloroplasts. Furthermore, plastid size heterogeneity was also observed in PCs and GCs of *atminE1* (Figs 2 and 4), in which mesophyll chloroplasts fail to initiate their division [27]. These phenotypes were exactly the same as those observed in *arc6* [7]. We cannot rule out the possibility that both ARC5/DRP5B and ARC6 act as plastid division site regulators in PCs and GCs and that, in these non-mesophyll cells, the role of MinE is opposite that in mesophyll cells (*i*.*e*., a MinD-like function). Nevertheless, a more plausible explanation is that correct division site positioning in plastids is more vulnerable to malfunctioning of the division ring machinery in non-mesophyll tissues than in the mesophyll. Perhaps such increased vulnerability is associated with the higher fluidity (or lower rigidity) of the plastid envelope membranes, as indicated by the more frequent formation of stromules compared with that of mesophyll chloroplasts. Under this scenario, the timing or extent of bulge and stromule development may contribute to the plastid heterogeneity detected in PCs. Our current and recent observations [28,63] strengthen the notion that leaf epidermal plastids tend to display high morphological variability on a cell-by-cell or even plastid-by-plastid basis, as induced by mutations in chloroplast division genes.

Concerning the developmental regulation of plastid morphology in the division mutants, we previously proposed that development-associated chloroplast expansion prevents the constriction events of the plastid envelope in leaf mesophylls of an *AtMinE1* overexpressor and *arc11*, a chloroplast division site positioning mutant [56,64]. Based on this, thylakoid development may be a critical index for distinguishing the plastid phenotypes of leaf epidermis and mesophylls. The thylakoid system itself has mechanical strength, which enables it to retain well-preserved forms following isolation from chloroplasts. Upon defects in the plastid division apparatus, epidermal plastids that are poor in thylakoid membranes might undergo division *via* a rudimentary FtsZ1 ring [28], whereas mesophyll chloroplasts tend to continuously grow because of restrictions caused by the presence or growth of their thylakoids. In support of this idea, a majority of FtsZ1 rings in epidermal plastids of *arc11* achieved plastid fission, unlike mesophyll chloroplasts [65]. Furthermore, it has been recently shown that thylakoid division in *Arabidopsis* mesophyll chloroplasts requires the chloroplast division machinery [66]. The relationship between thylakoid organization and plastid division is almost unknown and will require substantial investigation in the future [54].

The differences in phenotypes reported for epidermal plastids in *arc5*, *arc6* or *atminE1* are an important consideration. In particular, in *arc6*, despite well-conserved GC structures, there have been some differences in the literature concerning the size, shape, and number of GC plastids (chloroplasts) [5,7,18,19]. As a whole, the GC plastids observed in the current study were more highly developed, and more chloroplasts were produced in the plant samples examined than in other studies. Differences in the study materials, such as the use of rosette leaves ([5,7,18] and this study) or cotyledons [19], the adaxial (this study) or abaxial [5,7,18] side of leaves, and soil [5,7,18] or solid medium (this study) for plant cultivation, may explain the variations in GC phenotypes among studies. As recently reported for the hypocotyl and root epidermal cells [67], differences in the distance from key proliferative zones for leaf development, such as the shoot apical meristem and the leaf blade–petiole junction [68,69], could also affect GC phenotypes. Furthermore, differences in organelle states could affect the physiological functions or activities of the GCs. The plastid morphology reported in this study should be of help in future studies investigating the roles of GCs in the control of stomatal opening and closing [70,71]. This would also be the case for studies designed to examine the roles of PCs.

## Supporting information

S1 Fig Plastid phenotype of *arc6* leaf PCs. A CLSM image of maximal intensity projection is shown. The arrowhead indicates two stromules that appear to align or wind together. See also Fig 2. Bar = 10 μm.
(TIF)

S2 Fig Distribution of YFP-labeled plastids in a GC pair in *arc5*. Serial optical sections of Fig 4D are shown. Bar = 10 μm.
(TIF)

S3 Fig Distribution of YFP-labeled plastids in GC pairs in *arc6*. (A, B) Serial optical sections of Fig 4F (A) and 4G (B) are shown. Bar = 10 μm.
(TIF)

S4 Fig Distribution of YFP-labeled plastids in a GC pair in *atminE1*. Serial optical sections of Fig 4I are shown. Bar = 10 μm.
(TIF)

S5 Fig Simulation of random GC pairing in *arc5*, *arc6*, and *atminE1*. For all 200 GCs, the number of each GC type is the same as the actual count in the mutant (Fig 5E). The GCs were randomly shuffled to create 100 GC pairs, and the number of each GC-pair type was counted in each simulation trial. The average counts from 1,000 trials for each mutant are shown (gray boxes), along with standard deviation (error bars). The actual counts of each GC-pair type in 100 GC pairs (Fig 5F) are also shown for each mutant (black boxes) for comparison. For *arc5*, bar plots for the low-count pairs are also shown, with a 9× magnified *y*-axis. The simulation program was written in Python 3.6 and is available upon request.
(TIF)

S1 Mov Three-dimensional image of YFP-labeled plastids in a GC pair in *arc5*. A three-dimensionally reconstructed image of the GC plastids in Fig 4J is shown with rotation around two axes.
(MOV)

S2 Mov Three-dimensional image of YFP-labeled plastids in a GC pair in *atminE1*. A three-dimensionally reconstructed image of the GC plastids in Fig 4K is shown with rotation around two axes.
(MOV)
