# Supplementary figures and images for: The Arabidopsis arc5 and arc6 mutations differentially affect plastid morphology in pavement and guard cells in the leaf epidermis
Source: PLoS One. 2018 Feb 21;13(2):e0192380. doi: 10.1371/journal.pone.0192380 (PMC5821325; doi:10.1371/journal.pone.0192380)

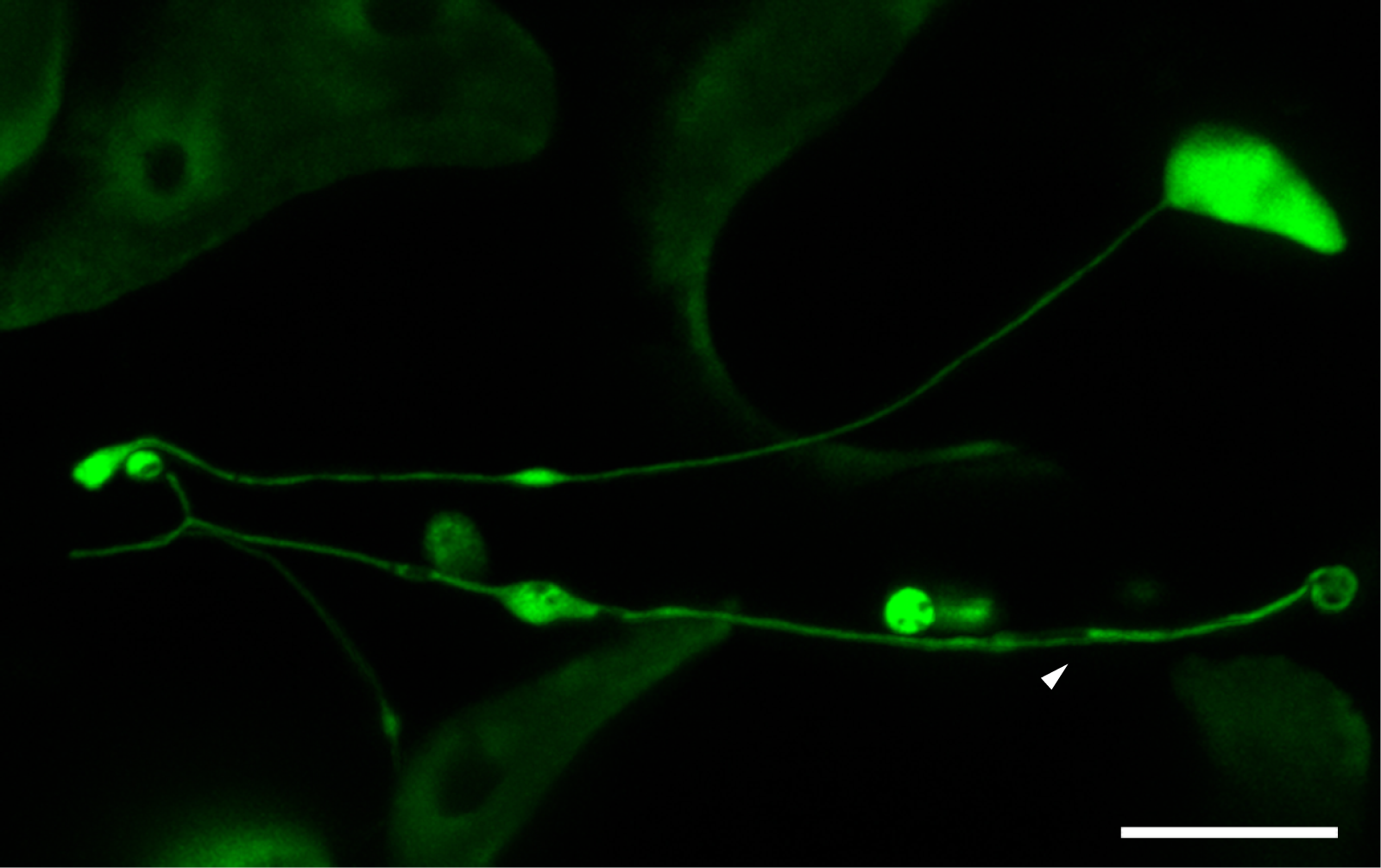

Supplement: S1 Fig — A CLSM image of maximal intensity projection is shown. The arrowhead indicates two stromules that appear to align or wind together. See also Fig 2. Bar = 10 μm. (TIF) [file pone.0192380.s001.tif]

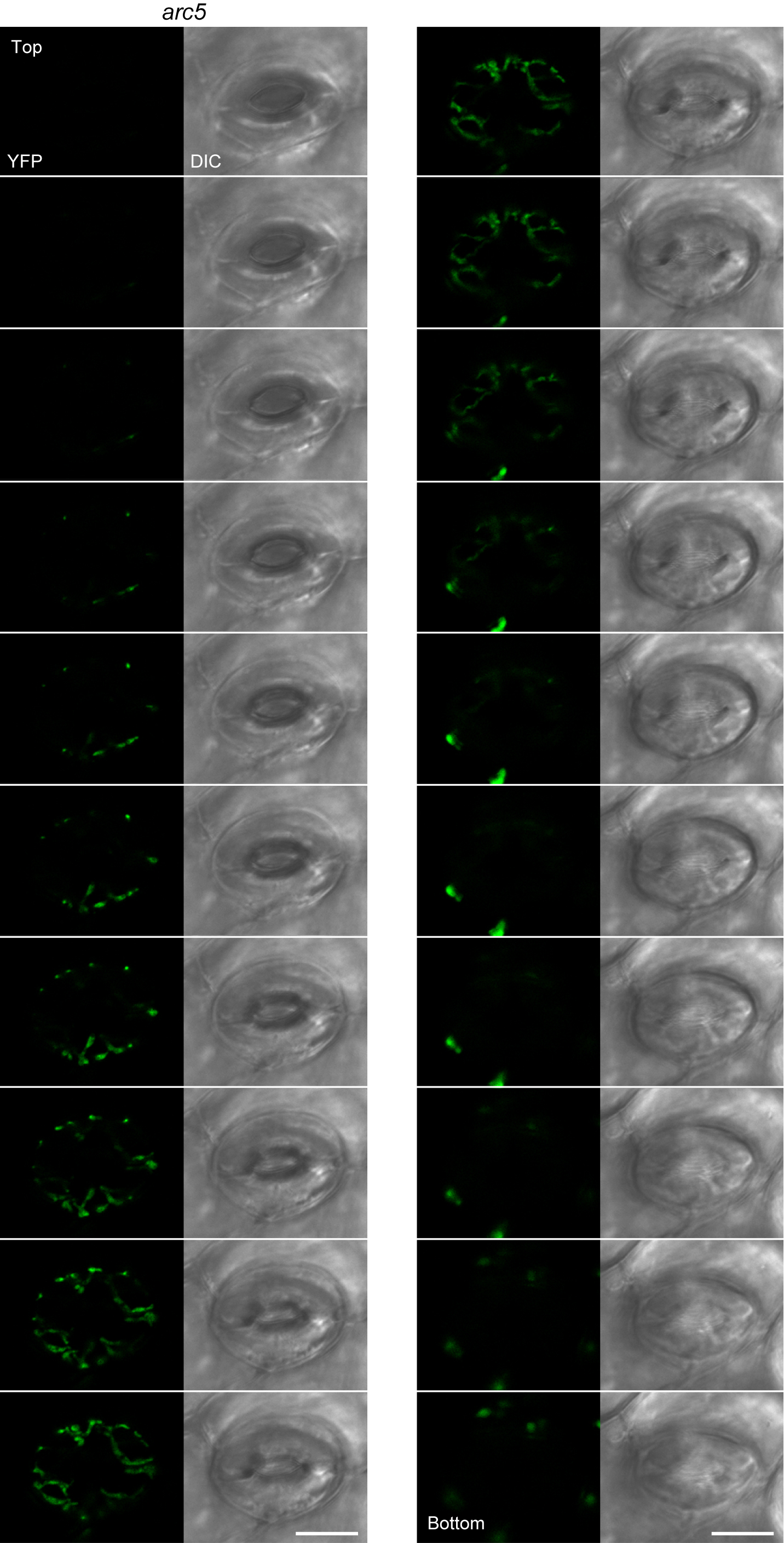

Supplement: S2 Fig — Serial optical sections of Fig 4D are shown. Bar = 10 μm. (TIF) [file pone.0192380.s002.tif]

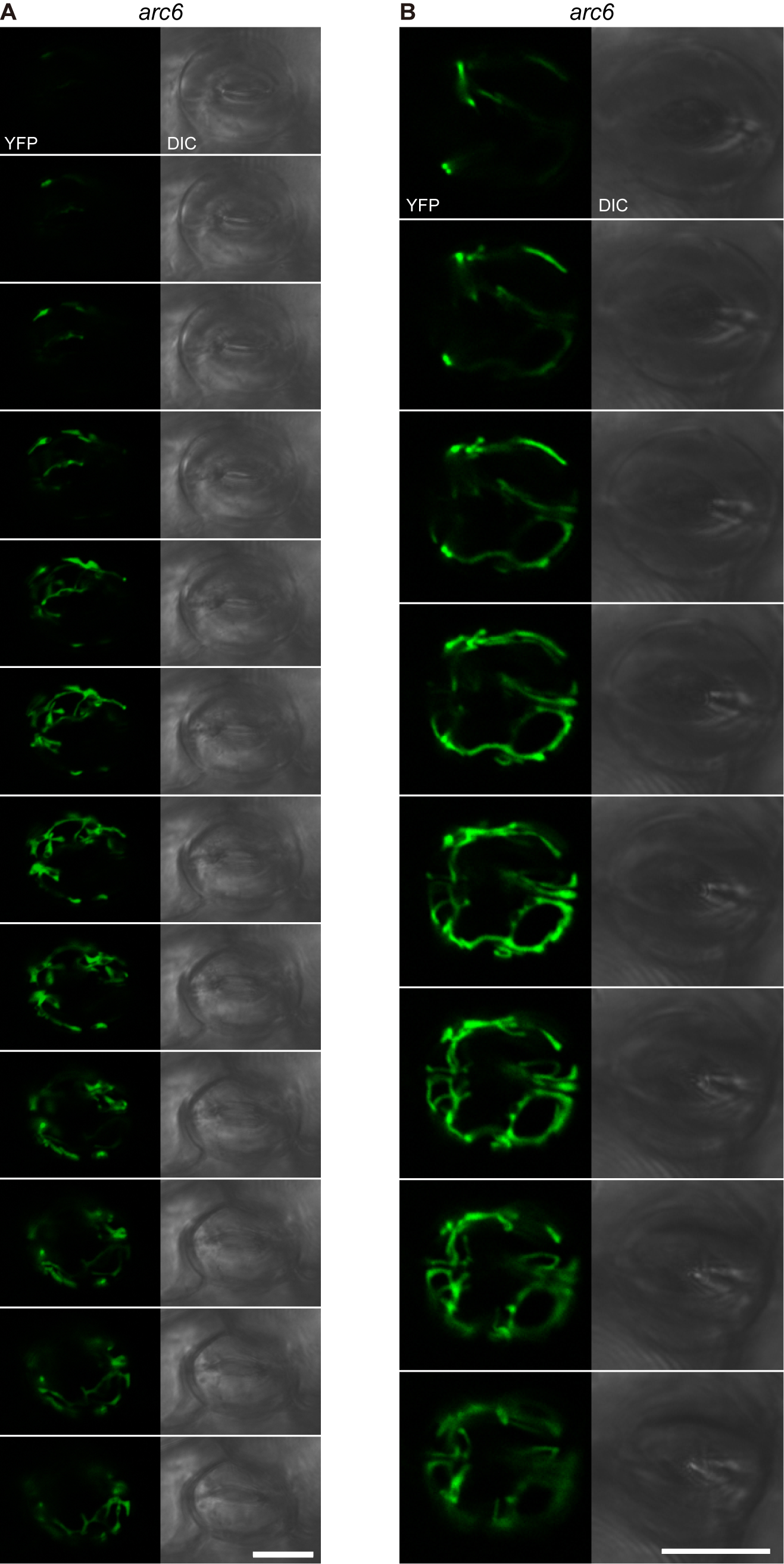

Supplement: S3 Fig — (A, B) Serial optical sections of Fig 4F (A) and 4G (B) are shown. Bar = 10 μm. (TIF) [file pone.0192380.s003.tif]

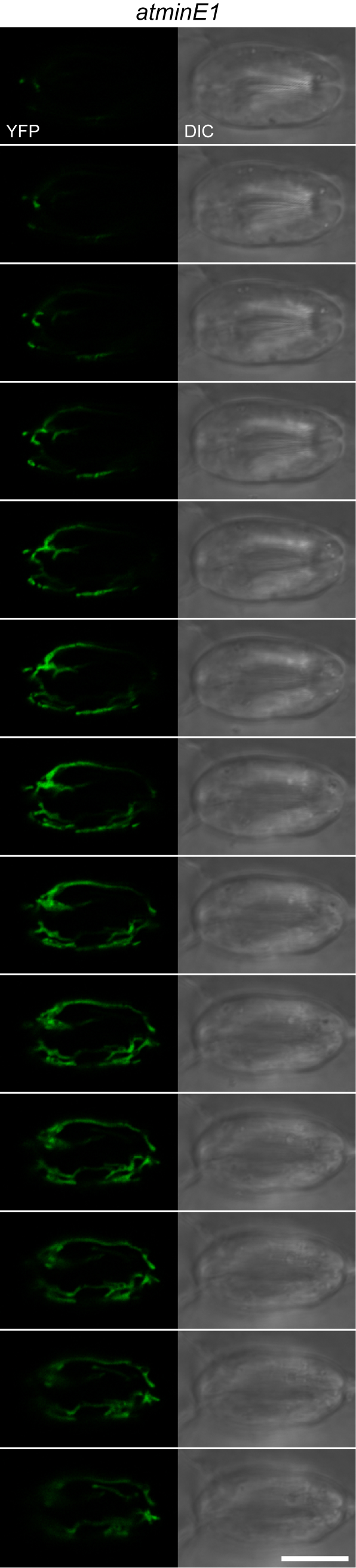

Supplement: S4 Fig — Serial optical sections of Fig 4I are shown. Bar = 10 μm. (TIF) [file pone.0192380.s004.tif]

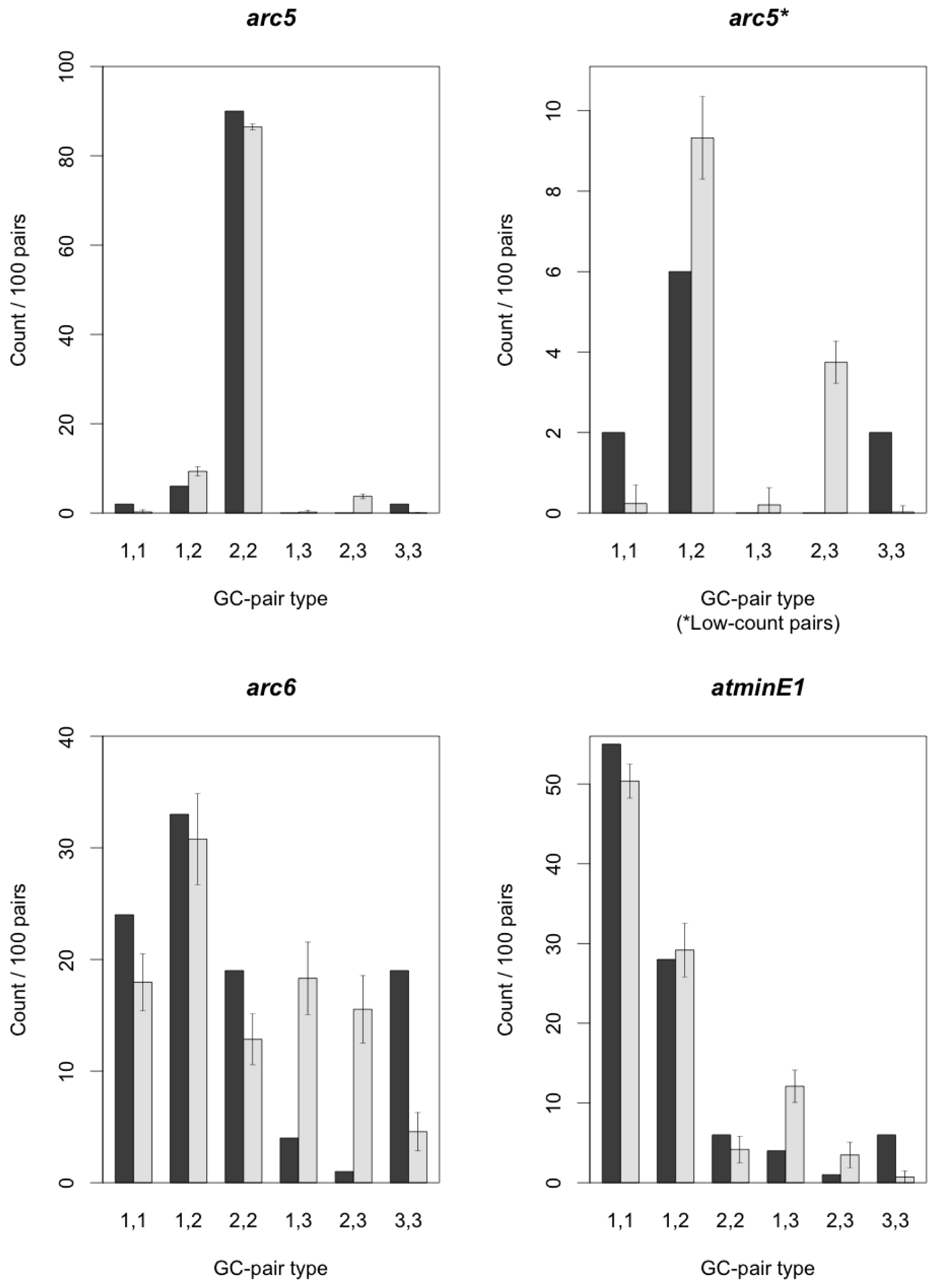

Supplement: S5 Fig — For all 200 GCs, the number of each GC type is the same as the actual count in the mutant (Fig 5E). The GCs were randomly shuffled to create 100 GC pairs, and the number of each GC-pair type was counted in each simulation trial. The average counts from 1,000 trials for each mutant are shown (gray boxes), along with standard deviation (error bars). The actual counts of each GC-pair type in 100 GC pairs (Fig 5F) are also shown for each mutant (black boxes) for comparison. For arc5, bar plots for the low-count pairs are also shown, with a 9× magnified y-axis. The simulation program was written in Python 3.6 and is available upon request. (TIF) [file pone.0192380.s005.tif]
